# Impact of COVID-19 on the lives of people with severe mental illness—front-line community psychiatry workers observation from a provincial survey of assertive community treatment teams in Ontario, Canada

**DOI:** 10.1186/s13033-023-00585-8

**Published:** 2023-06-16

**Authors:** Aly Kassam, Michaela Beder, Saadia Sediqzadah, Matthew Levy, Madeleine Ritts, John Maher, Nicole Kirwan, Samuel Law

**Affiliations:** 1 MAP Centre for Urban Health Solutions, Unity Health Toronto, Li Ka Shing Knowledge Institute, St. Michael’s Hospital, Toronto, ON Canada; 2 Department of Psychiatry, Faculty of Medicine, University of Toronto, Toronto, ON Canada; 3 Department of Psychiatry, Unity Health Toronto, St. Michael’s Hospital, Toronto, ON Canada; 4 Canadian Mental Health Association, Barrie and Ontario Association of ACT and FACT, Barrie, ON Canada

**Keywords:** Observational study, Observed impact, Community mental health, COVID-19, Serious mental illness, Assertive community treatment

## Abstract

Using an online survey distributed to members of the provincial organization that represents the 88 Assertive Community Treatment (ACT) and Flexible ACT teams in Ontario, Canada, this descriptive study relied on the unique vantage points and observations of the front-line community psychiatry workers who maintained contact with patients through outreach and telecommunication during the height of COVID-19. The patients who suffer from serious mental illness (SMI) were uniquely affected by COVID-19 due to the changes, reduction or shut down of many essential clinical and community support services. Thematic and quantitative analyses of the workers’ observations highlighted 6 main areas of note, including significant social isolation and loneliness, clinical course deterioration and life disruption, increased hospital and ER use, police and legal contacts, and substance abuse and related deaths. There were also encouraging signs of positive adaptations in terms of independence and resilience. Reflections of these impacts and potential ameliorating approaches are further discussed.

## Introduction

The COVID-19 pandemic has had unprecedented negative impact on the mental health of the general population [1]. More specifically, the impact on the lives of those already suffering from severe mental illness (SMI) is heavily anticipated and research has pointed to a disproportional and substantial impact on their health and safety, with preliminary data showing increased rates of depression, anxiety, and exacerbation or new onset of psychosis, among others [2–5]. They are also likely suffering from higher rates of physical illnesses [6, 7], a relative lack of insight and judgment on infection control practices and techniques, less self-care capacity, and fewer coping resources [8–11].

The more indirect impact on the lives of those with SMI is less known. The likely stressors include multiple and rapidly evolving infection control measures, social isolation, loss of resources and social support, and generally living in a community with heightened intensity of fear. These indirect impacts are insidious, often less readily evident and therefore easily neglected, yet carry long term effects. For example, even the most basic infection control strategy of social distancing may have heightened impact on those with SMI, as it compounds the social isolation and loneliness that are already prevalent in this population [12]. They are more likely to be living alone or with unrelated adults, and to have limited support systems or reduced autonomy [13]. Furthermore, reduction or closure of supportive resources such as drop-in centers, community kitchens, libraries, places of worship, and recreational centers also critically affect people with SMI who depend on these services for social support, community participation, and being socially included.

Moreover, community psychiatry as a field is often less prioritized in pandemic response planning, support, and evaluation [14]. In this sector, the Assertive Community Treatment (ACT) teams are unique, as they are often seen as the “gold standard” of care, serving almost exclusively those with SMI [15, 16]. ACT services are the most intensive form of psychiatric care, employing an outreach-based model, with a 24/7 on-call system, daily team meetings and home visits, a multidisciplinary team (including psychiatrists, nurses, social workers, occupational therapists, addictions counsellors, and peer support specialists), and a low staff-client ratio [17, 18]. ACT workers have a very intimate front-line perspective on the lives of those they serve, particularly given that most ACT teams have adjusted their care in order to maintain a large part of their outreach and close contact with their patients as part of their essential services during the pandemic [19, 20].

There is a unique opportunity to learn from ACT team workers on the pandemic’s impact on the lives of people with SMI through their front-line observations. While it would have been most ideal to study the patients directly, observations from experienced informants could also be a valuable research method [21], particularly in times of need for rapid identification of concerns, producing useful sentinel information that could guide clinical responses, understanding, and future research direction [22].

To further understand the impact of the COVID-19 pandemic on the lives of people living with SMI, we report the results of a province wide survey that captured observations of representative front-line staff of the 88 ACT and Flexible ACT (FACT) teams in Ontario, Canada. This indirect observed perspective form part of the overall understanding of this challenging population. Ontario is Canada’s largest province, with a population of 14.8 million [23]. Ontario has registered 1.45 million COVID-19 cases and 14291 deaths as of Sept 21, 2022 [24].

## Methods

The current observational study is part of a larger project that studied how ACT services and structures have changed and what adaptations and innovations were made in ACT and FACT teams during the pandemic. The study employed a survey that was developed by the research team, composed of academic and practicing psychiatrists and clinicians in community psychiatry. The survey was converted to an online questionnaire hosted by the Simple Survey platform (Outsidesoft Solutions Inc., 2022), and piloted with the research team to provide further feedback on its ease of use, clarity, length, and acceptability. A typical survey took 20–40 min to complete.

Using a convenience sampling method, the survey utilized the current email list of the Ontario Association of ACT and FACT (OAAF), the sole provincial organization that engages Ontario’s ACT and FACT teams in standard setting, fidelity and quality improvement, professional education, annual conferences, and political advocacy [25]. The inclusion criterion was anyone who was on the current mailing list of the OAAF. The survey was sent to all 232 individuals on the list, who typically consisted of 2–4 representative members per team, often team leaders (typically of Registered Nurse (RN), or Masters of Social Work (MSW), or Occupational Therapist (OT) backgrounds, senior members (typically with more years of experiences in community psychiatry), and managers (typically with both clinical and managerial experiences and similar education background as above) of the 88 ACT and FACT teams across Ontario. Each potential participant was sent a copy of the study information and consent form, and a link to the Simple Survey on April 12, 2021. Four reminder emails were sent out over the course of five weeks. Response collection closed on May 31, 2021; the surveyed period included part of the height of the Delta variant “third wave” COVID-19 pandemic [24]. All questions on the survey were optional, and responses were collected anonymously and respondents could save their answers to be completed later. Only completed and submitted responses are included in this analysis.

**Fig. 1:**
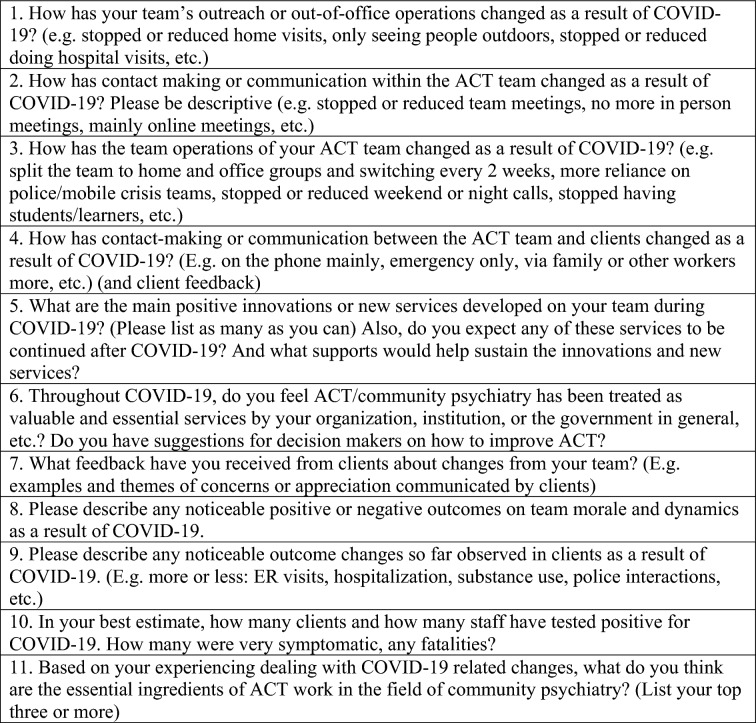
List of the 11 open-ended qualitative questions

The on-line survey consisted of a total of 40-questions: one basic demographic question that only solicited geographic and nature of services (ACT vs FACT) to ensure maximal anonymity, 28 Likert 5-point scale questions that reproduced the Dartmouth Assertive Community Treatment Scale (DACTS) [26] that measured how the fidelity of ACT teams changed during the pandemic in terms of human resources, organizational boundaries, and nature of services domains (results reported elsewhere), and 11 open-ended questions that dealt with team service and functional changes, observations on the impact in daily lives and clinical outcomes of the clients, and feedback from clients to the team, as observed and reflected upon by the ACT/FACT workers. The data may capture what the workers have heard from patients directly, but it is chiefly observational and reflective. The respondents were given unlimited space to write their answers in the survey. Typical answers were in sentences and some in point forms. The current study uses data from answers to these the 11 qualitative questions.. A sample of these questions included: *What are the main positive innovations or new services developed on your team? What feedback have you received from clients about changes from your team? Please describe any noticeable outcome changes so far observed in clients,* etc. (see Fig. 1).

The answers to the open-ended question were processed and entered into NVivo 12 software (QSR International, 2021). Research team members (AK & SL) used thematic analysis methodology, with an inductive reasoning approach and analyzed the data by creating memos, notes, and queries, and identified themes that were commonly repeated. The results were verified by the rest of the research team [27]. In analyses of the data, the researcher team was informed by, among others, the classic conceptual domains of quality of life, issues such as clients’ sense of well-being, relationships with clinicians and family, recreational activities, personal development, and general independence and adaptations [28]. Selected search terms related to clinical and social outcomes and quality of life were decided upon by the research team and all occurrences of each term, including exact match, stemmed words, and synonyms (which were automatically selected using NVivo’s query page), were recorded.

This study was performed in accordance with the Declaration of Helsinki for human subject research, and approved by the St. Michael’s Hospital, Unity Health Toronto Research and Ethics Board.

## Results

The final data set consisted of 144 completed (62.1%, of 232 sent) surveys. The responses were representative of the province, with 32 responses from Metro Toronto, the provincial capital, 57 from large population centers (1 million + excluding Toronto), 29 from medium sized (30,000–99,999) population centers, 20 from small (< 30,000) population centers, 4 from rural regions, and 2 of unknown origin. We used simple descriptive statistics of percentages of respondents having mentioned a particular key term or notion to illustrate the popularity and salience of a finding. The following are six major themes of the findings (Of note, the survey showed that, up to the point of the closing of the survey, there were at least 6 reported fatalities related to COVID-19, 2 other deaths that were complex and of unclear relationship to the COVID-19 pandemic, and about a dozen or so patients who tested positive for COVID-19).

1. Significant increase in social isolation

Isolation was reported 38 times (26.4%) in the survey, making it a leading theme of the survey. There were multiple facets related to the reported social isolation. One respondent explained*: “[They have] difficulty to maintain face to face contact to break isolation”.* Another elaborated: “*We have seen a decrease in physical health of several clients who were previously stable and it is unclear if this has to do with the length of time they have been isolating with less support.”* More commonly, with clients avoiding coming to the clinic or seeing family or friends out of caution, or fear of contracting COVID-19, one respondent described: *“Some clients becoming more isolated and reclusive i.e. refusing even essential in person doctors* [sic] *appointments. Fear of exposure from staff by clients and families”.*

Beyond isolation, some clients may have become avoidant, causing a threat to health, as one clinician noted: *“The change in routine due to Covid-19 had a negative impact on some clients' wellness—since they were no longer able to participate in group activities, run errands, see family or friends, take public transit, *etc*., in some cases there has been more difficulty in getting clients to hospital when they are very unwell in the community”.* One worker observed further connection between loss of support, social isolation and life and health changes: *“Clients suffer from community programs not being available, increased anxiety, low mood due to isolation”.* Another worker simply said:* “Increasing boredom* = *sadness due to Covid…”* More sobering, one observer reported: “*Some clients have had loss of loved ones during this time. Increased isolation and difficult to get some clients to engage*”. And in one extreme case: “W*e had a client suicide as a result of isolation and not being able to do their volunteering which brought meaning to their life…”* In the survey, 5 (3.5%) respondents noted an increased rate of client suicide crises during the pandemic.

2. Notable negative impact on daily lives and clinical courses related to community resource changes

Due to lock downs and infection control protocols, there were many unmet needs and difficulty in obtaining services reported (18.6%), ranging from a lack of housing services, food banks, drop-ins, to detox centers, in–person medical clinics and hospital beds. One worker reported housing challenges: “*As clients are getting displaced for not ‘following the rules’ and putting others at risk, there was nowhere for them to go without leaving the area that they reside and have resources or family”.* Another said: “*Difficulties obtaining a shelter bed due to reduced capacity in the system".* Related to hospital bed shortage, one respondent even suggested a link to three client deaths: *“As it was hard to support clients if they are homeless, don’t have a way to communicate (no phone) or are being transferred from hospital to nursing homes (during the start of pandemic). If clients could have stayed longer in hospital maybe we wouldn’t have had those three deaths”*. Another said: *“Of those who died, they were more lonely and had almost no other supported services from other community services”.*

As the pandemic and regulations continued, the respondents observed that clients’ perceptions changed. At the start of the pandemic (March–May, 2020) clients were more likely to be adherent to stay-at-home regulations and wearing of personal protective equipment, but over time they would become more “*bored*”, “*lonely*”, “*anxious*”, and “*stressed*”. An example of clinical impact was described by a staff: *“Medication compliance has decreased for several clients when face to face contacts have been decreased. This has led to several clients’ overall level of functioning decreasing for significant periods of time”.* One worker added: “*Trend of clients who have been mentally well for a long period of time are now going off of medications and needing to be hospitalized”.*

Additionally, some staff reported the halting or loss of long-fought gains in rehabilitation: *“The biggest noticeable negative impact is clients that had momentum for rehab goals whether it would be social groups, employment, exercise as it takes a lot of interventions to get them started prior. With Covid everything came to a halt therefore it's like also they have come to a halt. The opportunities have decreased.”*

3. Increased use of Emergency Rooms and hospitals and corresponding loss of in-person community mental health services

Increased hospital and Emergency Room visits has also been a major theme (29.9%). Multiple respondents stated that some clients were making more trips to the hospital simply to have face-to-face contact with someone, as one said: “*One year into the pandemic, we see more ER visits, clients appear starved for attention because they don't get the supports they need and we have seen overuse of hospital services for events that are not deemed "crisis".* Another worker offered*: “I can speculate that some of the hospitalization have been from increased isolation and/or substance use”.* One worker also observed: *“Some clients’ ER visits have increased [because they] have relapsed to substance abuse”.*

The increase in hospital or Emergency Room visits corresponded to the reported major changes in services: shifting towards virtual care (phone or online) (> 50%), reduced in-person clinics (40.3%), reduced frequency of home visits (47.9%), hospital visits (22.9%); moving community visits outdoors (42.0%); and reduced social services (58.3%). Quality of life enhancing activities such as helping clients in transportation, accompaniment to medical appointments, grocery shopping, etc. were largely lost. The negative impact of these changes on the clients were notable, as one participant described: *“Contacts are often very similar at each contact and it makes me wonder what good we are even doing. It also makes me question whether these contacts are at all helpful for the client because the decreased quality of contacts…, [they do] not appear to be supportive, especially for those who lack insight into why their workers cannot see them in person*”. Furthermore, some clients were homeless or very poor and have no access to phones, losing out on the changed services altogether. One staff also noted: “*We have discovered there is a lack of literacy regarding technology among our clients as well as lack of trust in the technology*.”

Other impacts on some of the unique areas of needs by people with SMI included cuts of visits to in-patient wards when they are hospitalized, or to jails when incarcerated. Sometimes the impact is more immediate, as one staff described: *“Increased homelessness and inability to access housing programs for a significant period. Inability to reach programs that assist clients with their budgets as they are working from home and only in office once week.”* One staff also reported the importance of contact and impact on the care of people with SMI: “*No new client intake during lockdown—need face-to-face visits to establish rapport.*”

In addition, the impact on socialization, educational and participatory activities were substantially affected—many respondents reported cancellations, decrease in frequency, switching to online groups, or reduction in size of group sessions (18.8%). One respondent observed that the clients had: “*Worsening quality of life as a result of not being able to connect with other groups*”. Amidst the multitudes of reductions, one staff summarized their visits and impact as: “*Many families stopped seeing our clients so we were the only ones visiting*”.

4. Increased medical-legal and police involvement

Another notable theme was increased contact with police and justice services (6.3%). As some of the outreach services were pared down during COVID-19, some treatment plans were interrupted and some contact were less timely, leading to safety and clinical concerns and risk of decompensation. To continue essential care, services often relied on Mental Health Act mandated mechanisms, based on legal Forms and involving the police or emergency response staff. This impersonal and legalistic approach has negative implications on the daily lives and relationships between clinicians and clients. One worker noted: “*There is a noticeable increase in hospitalizations and use of Form 47* [a Form to bring a patient involuntarily into hospital based on a Community Treatment Order or Out-patient Commitment legislations] *or Form 1* [Mental Health Act-based Form to involuntarily bring a patient by the police to hospital for psychiatric assessment]*. We don't ‘catch’ the clients in time when we can't assess them in person on a regular basis (some are very good to hide the symptoms during a phone call)”.*

There was also increased reaching out to Mobile Crisis Intervention Teams (i.e. MCIT, where a mental health nurse is paired with a police officer to address emergency phone calls related to mental health) to find those who are hard to reach or too unwell, as one staff reported: “*On occasion there has been some increased reliance on mobile crisis services for our unhoused/under-housed folks*.” Another observed: “*We definitely rely on police/mobile crisis more now and have been much less involved in all crisis hospital visits for clients—sometimes they even get to hospital and we are not involved in the transfer to a schedule 1* [i.e., accredited general hospitals where patients can be admitted under a Form 1] *facility*”. The pandemic has caused an interconnected vicious cycle of life changes, one worker summed it up as: “*More ER visits, more substance use, more police interactions, more justice involvement …more instability*”.

5. Substantial increase in substance use

Upsurge of substance use from pre-pandemic levels among the clients with SMI was another major theme (31.1%). The respondents were very straightforward in their report, as one simply said: “*More and more…substance use* +  +  + ”. Some also observed consequences: “*More substance use and police interactions*”; “*Increased substance use and more ER visits*”; and “*There has been an increase in drug use and homelessness and ER visits have increase* [sic] *with clients who haven't utilized as much before*”. A parallel problem with negative impact was also: “*It has been much harder to find clients appropriate shelter and substance use treatment due to the Covid-19 pandemic*”. Many also offered speculations and theories on the rise in substance use: “*Clients are bored, more substance use*”; “*More use, more anxiety observed in clients and feeling isolated*”; “*Substance abuse has increased along with decreased participation in group and ability to effectively educate from solely a virtual platform*”.

Most concerning, staff have found: “*For some, increased or more risky substance use*”; and “*Drug overdose and accidental death has increased*”. There were multiple reports of opioid related deaths, as one accounted: “*It seems that the opioid crisis has exploded during the pandemic and morale is affected…”* One respondent said one such death was due to the client’s: “*Reluctance to visit the ER, potentially due to concerns about Covid -19”*.

6. Positive adaptations, and emergence of independence and resilience

There were also many reports of positive adaptations in the face of COVID-19 (13.19%). A few respondents mentioned that their clients had actually reduced hospital/ER visits, police/authority encounters, or substance use, but these responses were in the minority. A number of workers reported that some clients thrived from the reduced social interactions and stay-at-home orders, as one staff said: “*The patients that were hard to get them to come to the office are loving that they get a phone call to address issues they may not have done if they were face to face with the doctor”.* And another felt: "*Virtual and phone are allowing clients to share freely. Many clients claim they feel more comfortable sharing feelings, thoughts, risks over the phone”.* One staff mentioned many positive outcomes: *“Some clients have improved, started a new job, moved into a new apartment *etc*. Some cts* [sic] *have been transferred out of the program to a less intensive case management service. Some have been discharged to the care of their primary care provider”.*

In addition, many noted increased independence of clients (23.6%). One staff noted an example: *“Not driving clients. Empowering clients to access transportation themselves *via* bus or with support *via* taxi or bus”.* Another added: *“A push for more client independence produced good results re: independent grocery shopping and use of transit system for transportation”; “Very proud of our clients and their coping strategies”.* One staff also noted: *“Many clients have demonstrated an increased accountability for their own healthcare for example we switched safe clients to monthly home medication delivery rather than weekly ACTT drop offs.”*

Overall, the impact of COVID-19 has produced welcomed changes in some, as one summarized: *“Greater emphasis has been placed on the client's independence and autonomy during their recovery journey from mental illness, instead of the dependence that can naturally develop on ACTT support”.*

## Discussion

Using a survey to gather information from community mental health workers who maintained contact and continued to work intimately with people with SMI to understand *indirectly* the impact of COVID-19 on their clients’ lives, the current study produced a concerning picture of social isolation, clinical course deterioration, life disruption, increased hospital and ER use, increased police and legal contacts, and increased substance abuse and related deaths. On the other hand, we have also witnessed encouraging signs of positive adaptations.

While the most common and sensible approach to study impact on patients’ lives would have been studying the patients directly, our study is a compromise using only the observer’s perspective. However, the study does carry some advantages as it can be timely, rapidly produced, and guide further more direct and detailed research during a very disruptive pandemic [22].

Our study also carries a more clinical perspective from our specific informants., T front-line workers have noted how much of the pandemic’s impact has been likely mediated through the loss of normal community mental health services [29], and how critical these services have been to the people with SMI [30]. This lends credence why high-standard community psychiatric care has centered around close contact, quick responses, and building and enhancing clients’ social and lived world through regular, in-person outreach-based support [18, 31]. In that context, the pandemic related reduction and withdrawing from such key support has posed a strong and unique challenge to this population. Overall, it is a strong belief within community psychiatry that their work in times of crisis is vital in providing continuity of care, a sense of hope and connection, and promoting resilience [32].

In terms of more specific impact, our finding on the pandemic related social isolation and loneliness experienced by the patients have been very notable. The pandemic may be particularly impactful for individuals with SMI as they are more likely to have low-pre-existing social support, suffer low social status and stigma, with limited coping skills and strategies to face the heightened emotional distress during the pandemic [33]. In general, loneliness is not only associated with poor mental health [34], but has also long been linked to poor physical well-being [35], lower quality of life, depression, suicide ideation [36], substance use [37], and possibly even psychotic symptoms [38, 39]. Research has also shown the social ties and virtual connection formed via telecommunication media are not as ameliorative as one might expect, making the pandemic-related shifting to virtual care less promising [40, 41]. Strengthening the coping skills and the ability to be at peace with oneself, improving social bonds with others, being sensitive to the detrimental effects of loneliness and tailoring services accordingly are some of the proposed solutions [42, 43]. Furthermore, as an extension of this theme, our study highlights the importance of social inclusion for people with SMI—a practice that promotes social participation, meaningful social supports, proper housing, neighborhood and community involvement, employment and education, and appropriate service utilization [44]. Social inclusion is associated with enhanced self-esteem, lower stigma, and improved overall neuro-cognition and quality of life, but is often under-recognized [45, 46].

Our observed impact on the clinical courses of the SMI clients in terms of clinical deterioration, and having increased hospital and ER visits is also remarkable. There is unfortunately very limited published evidence or quantitative data to date to support these observations. Research has shown mental health in the general populations has worsened during the pandemic, with a two- to three-fold increase in mood and anxiety disorders [47]. A Scandinavian online study on out-patients with prior mental illness shows 52% of the respondents reported deteriorated mental health during the pandemic, while 33% reported no change, and 16% reported improvement. The most commonly cited reasons for their deterioration were loneliness, disruption of routines, concerns regarding the coronavirus, less contact with family/friends, boredom, and reduced access to psychiatric care [48]. These were remarkably similar to those observed by our respondents. However, little is known about people with SMI more specifically, but experts have widely sounded concern that patients with SMI are extremely vulnerable to relapsing during the COVID-19 pandemic when compared to the general population [3], and condition such as anxiety, depression, panic, delirium, psychosis and suicidality are expected to worsen [49]. One qualitative US study has found that patients with SMI reported feeling overwhelmed and distressed, had difficulty concentrating, concerned about medical bills, missing appointments and having enough food, and significantly increased tobacco use [50]. In this vein, the current study’s early front-line observations are valuable as confirmatory data and possibly a harbinger of what has happened and what may come next so one can be informed towards more immediate response to improve the situation.

The clinical changes observed by the study may in parts reflect the decrease in ACT services during the pandemic, how clients with SMI seek help in ways they know how, the stress of the pandemic, the closure or reduction of usual community and general medical services, and other more common factors such as non-adherence to treatment, poor social and cognitive functioning related to SMI, substance abuse, and social isolation [11, 34]. The reasons for the clinical deterioration are undoubtedly complex and interactive. For ACT teams, the most well-known metric measuring outcome is the number and duration of client hospital visits. To that end, the pandemic’s impact is quite evident by observation, despite having no systematic empirical data to support the observation yet. Looking at what factors that had changed within community psychiatry, one will likely conclude that the in-person contacts, built-in psychosocial services, group activities, and clinical treatment are very important for client stability [51].

Of the other many major disruptions and impact on the daily lives of individuals with SMI, one notable finding is the lack of housing and shelter. Likely similar in many other jurisdictions, Ontario has struggled with an underfunded community mental health sector, and poor access to affordable housing for people with SMI [52]. During the pandemic, these preexisting inequities and disparities have evidently been amplified [53, 54].

We have also found an increase in police and emergency response contacts. Canadian estimates vary, but normally up to 18.8% of police calls are related to mental health issues [55], making them part of the front line mental health workers [56]. Our study has underlined this phenomenon even more acutely in the time of the pandemic, as more calls for help, and Mental Health Act related apprehension orders are placed with the police and emergency response services [57]. When an ACT service that is known to cherish in-person contact, and developing close rapport with their clients has to resort to a legal mandate to bring patients to hospital, it is unfortunate and usually a last resort, as there is often negative impact that includes loss of trust, rapport, and therapeutic alliance [58, 59]. The increase in police contact is also most likely related to the complex interactive picture that goes beyond the reduction of normal services, but involving clinical deterioration, more substance abuse related dysregulation, and difficulty finding help at a time of social distancing. It is unfortunate that the police had to shoulder a sizable part of the burden in the time of a pandemic, but they have been some of the under-recognized essential service providers [60].

Our study also observed increases in substance misuse, often with more addictions related services utilization, police and ER encounters, and at times, fatal consequences. Furthermore, there was limited access to detoxification centers and addictions services during the pandemic, associated with additional distress and dire consequences. In general, co-occurring addictions issues in individuals with SMI is elevated when compared to the general population [61, 62]. One US national survey estimated that 49.4% of adults with SMI used illicit drugs, compared to 15.7% for the national average [63]. Reasons for such elevated use are thought to be commonly related to this population’s general need to cope with stress, anxiety, depression and other psychiatric symptoms, dealing with side effects of medications, relative lack of insight and judgment, poor coping skills, and ambivalence towards substance use at large, among others [64, 65]. In the time of the pandemic, these same reasons are consistent with this study’s front-line reports and they are even more intensified, paralleling the global reports of increased substance use and other deleterious health behaviors during the pandemic [66–69]. Furthermore, at least locally in Ontario, research shows that there is a concurrent crisis of an opioid epidemic within the COVID-19 pandemic [70]. The current survey has given a disturbing glimpse of the impact the pandemic has had on substance use that seemed to over shadow the pandemic morbidity itself. Our findings also point to the need to ensure addictions care are available, and emergency responses and preventative policies are urgently needed [71, 72].

At a broader level, while the pandemic’s impact on the clients can seem both clinically specific, and wide ranging, a helpful way to contextualize these impacts is to use an objective quality of life framework [73]. The current study provides evidence that many classic domains related to quality of life were disrupted and affected, particularly in the clients’ daily routines, housing stability, clinical support, community and supportive activities, social participation, and mental and physical well-being [74]. In other words, the very negative external life conditions could often impair the global subjective wellbeing for most, as previous research has demonstrated [75]. Another pathway that affects one’s quality of life is mediated through unmet needs. With the pandemic, many services and resources were shut down, most likely contributing to the deterioration of clients’ quality of life [76]. Furthermore, the loss of the usual positive and supportive social interaction with clinicians or family may play an out-sized role for people with SMI as they often encounter negative, stigmatized interactions in their routine lives, making the loss or reduction of their positive socializations particularly impactful [77], as one worker observed that workers are often the only “family” the patients have.

On a brighter note, our study has found that the pandemic has also led to more self-efficacy and independence in some clients. This is inspiring for furthering the recovery-oriented model of care, built on acceptance, optimism, shared decision-making and tailored support [78, 79]. Research has shown that a recovery-oriented care model is associated with lower hospitalization days, less legal involvement, and enhanced education achievement and employment [80], as well as enhancing mastery, autonomy, self-efficacy, self-esteem, and quality of life [81], and building resilience [82]. Whether the observed achievements in the clients during the pandemic reflect a more recovery-oriented practice in community psychiatry remains to be better evaluated, but our observation may be one positive, affirming lesson we have learned from the pandemic.

The limitations of the current study include the fact that it is an indirect observation, absent patients’ direct input, affecting its validity. Further research with patients more directly would be valuable. It is also a conveniently sampled survey of one particular jurisdiction in Canada, and cross-sectional perspectives over a finite period of time within a protracted pandemic. While it aims to provide a broad observer perspective on an important question, the generalizability of the observed and reflected results are limited. No causal relationship is possible for the observed data. The response rate was moderate and may be biased as those who were more inclined to share positive findings or those who were more dissatisfied may be over-represented. Further research as informed by these preliminary and observational results is highly warranted.

## Data Availability

Original survey data and analysis are available on an as needed basis.
